# Participatory development and pilot testing of *i*Choose*:* an adaptation of an evidence-based paediatric weight management program for community implementation

**DOI:** 10.1186/s12889-019-6450-9

**Published:** 2019-01-29

**Authors:** Jennie L. Hill, Jamie M. Zoellner, Wen You, Donna J. Brock, Bryan Price, Ramine C. Alexander, Madlyn Frisard, Fabiana Brito, Xiaolu Hou, Paul A. Estabrooks

**Affiliations:** 1 Department of Epidemiology, College of Public Health, University of Nebraska Medical Center, Omaha, USA; 2 Department of Public Health Sciences, School of Medicine, P.O. Box 800717, Charlottesville, VA 22908-0717 USA; 3 Department of Agricultural and Applied Economics, Virginia Tech, Blacksburg, VA 24061 USA; 4 Education and Outreach Specialist, University of Virginia and Cancer Center, P.O. Box 800717, Charlottesville, VA 22908-0717 USA; 5 Department of Family and Consumer Sciences, North Carolina Agricultural and Technical State University, Benbow 202-A, Greensboro, NC 27405 USA; 6 Department of Human Nutrition, Foods and Exercise, Virginia Tech, 1981 Kraft Drive (0913), ILSB 23, Rm 1085, Blacksburg, VA 24061 USA; 7 College of Public Health, University of Nebraska Medical Center, 984365 Nebraska Medical Center, Omaha, NE 68198-4365 USA; 8 Virginia Tech University, Blacksburg, USA; 9 Department of Health Promotion, College of Public Health, University of Nebraska Medical Center, 986075 Nebraska Medical Center, Omaha, NE 68198-6075 USA

**Keywords:** Childhood obesity, Evidence-based programs, Community-based participatory research, Program adoption

## Abstract

**Background:**

To describe the identification, adaptation, and testing of an evidence-based pediatric weight management program for a health disparate community.

**Methods:**

A community advisory board (CAB) of decision-makers and staff from local health care, public health, and recreation organizations engaged with academic partners to select an evidence-based program (EBP) for local implementation. Three EBPs were identified (Traffic Light, Bright Bodies, Golan and colleagues Home Environmental Model) and each EBP was rated on program characteristics, implementation and adaptation, and adoptability. Following selection of the EBP that was rated highest, the POPS-CAB made adaptations based on the program principles described in peer-reviewed publications. The adapted intervention, *i*Choose, was then pilot tested in 3 iterative phases delivered initially by research partners, then co-delivered by research and community partners, then delivered by community partners. The RE-AIM framework was used to plan and evaluate the *i*Choose intervention across all waves with assessments at baseline, post program (3 months), and follow-up (6 months).

**Results:**

Bright Bodies rated highest on program characteristics and adoptability (p’s < 0.05), while Home Environmental Model rated highest on implementation factors (*p* < 0.05). Qualitatively, the selection focused on important program characteristics and on matching those characteristics to the potential to fit within the community partner services. The adapted program—*i*Choose—had 18% reach and with participants that were representative of the target population on age, gender, ethnicity, and race. Effectiveness was demonstrated by modest, but significant reductions in BMI z-scores at post-program compared to baseline (M_Δ_ = − 0.047; t = − 2.11, *p* = 0.046). This decrease returned to values similar to baseline 3 months (M_Δ_ = 0.009) after the program was completed. Implementation fidelity was high and implementation fidelity did not differ between community or research delivery agents.

**Conclusion:**

The process to help organizations identify and select evidence-based programs appropriate for their community led to consensus on a single EBP. While *i*Choose was successful in initiating changes in BMI z-scores, could be implemented in a low resource community with fidelity, it was insufficient to lead to sustained child BMI z-scores. In response to these data, maintenance of program effects and delivery are the current focus of the CBPR team.

## Background

A large body of literature suggests that efficacious intervention strategies are currently available for treating childhood obesity [1]. In particular, family-based interventions that target the parent, or the parent and child, have efficaciously reduced and maintained child weight status [2–10]. For example, Epstein and colleagues developed the Traffic Light model which first demonstrated efficacy over 30 years ago and includes an explicit method for reducing caloric intake, increasing the intake of more healthful foods, and decreasing the intake of less healthful foods [11]. Golan and colleagues developed a health centric approach that focused on changes to the home environment that a parent can make to improve the likelihood that a child will eat better and be more active. In contrast to the Traffic Light program that includes contact with the parent and child, Golan’s model is a parent-only program [6]. Finally, Bright Bodies developed by Savoye and colleagues, provided a balanced program to support parents and overweight children in addressing energy intake and expenditure [10]. From a pragmatic perspective, each of these programs varies in implementation appeal, based on the number and duration of contacts, contact targets (i.e. parent and child vs. parent only), and associated implementation costs. Impressively, each of these programs includes data that demonstrates the reduction in childhood obesity can be sustained, and in some cases improved further, well after the initial intervention is complete [6, 8, 12, 13].

Unfortunately, there is little evidence that these childhood obesity programs have been systematically translated into regular practice [14–16]. It is possible that the same features of childhood obesity programs (e.g., high frequency and duration of contact; multi-disciplinary team) that lead to efficacy are also those that reduce the likelihood of adoption. For example, a recommendation in the journal *Pediatrics* included the need to have interventions that consisted of a minimum of 26 to 75 contact hours with participants [17]. This recommendation may be daunting for community and healthcare organizations in general, but is even more challenging within organizations that provide services to low-income families. Further, these efficacious programs rely on a multi-disciplinary team of experts including a paediatrician, registered dietician, and behavioural and exercise specialists, expertise that may not be readily available to all segments of the populations. Unsurprising, the most efficacious paediatric obesity treatment interventions have been based in urban areas delivered through large hospitals or medical centers where this type of expertise is readily available within the system [18]. The recent childhood obesity treatment recommendations do not address geographically underserved audiences or settings where all members of a multi-disciplinary team may not exist -- either within one organization or within the community or region.

An emerging body of literature suggests that systems-based approaches may be ideal to support the identification, adaptation, and implementation of locally relevant, evidence-based childhood obesity programs [19–24]. Indeed, the key to success for childhood obesity implementation may be the presence of strong and engaged healthcare and community partners [25]. Community-based participatory research (CBPR) partnerships are ideal settings for achieving horizontal engagement across local organizations, a key to systems based approaches [26, 27]. CBPR is a process that builds equitable community-academic partnerships and involves the community in all phases of the research process including assessment of the problem, identification and selection of potential interventions for the community, planning and development of the intervention, as well as monitoring and evaluation [28]. Although systems-based and CBPR approaches hold promise in identifying, adapting, and implementing evidence-based childhood obesity programs, studies to date have focused on prevention [29–31] or treatment focusing on engaging parents and not systems in the participatory process [32, 33]. This is a significant gap in the literature given that evidence-based program selection is identified as an important step in the implementation process [19, 34–37]. It is difficult to engage community members in all aspects of a research project without addressing practical methods to help partner organizations review, rate, and select an evidence-based program for local implementation. Further the degree to which local partners (e.g., public health departments/ youth-serving organizations like Parks and Recreation) could be engaged to adapt and implement components of an obesity treatment program in small cities or rural areas is unknown. Addressing this is a critical question for medically-underserved areas where the health professionals who typically deliver childhood obesity treatment programs are not available (e.g., behavioural counsellor; registered dietitian).

The purpose of this paper is to describe the steps taken to identify, develop and preliminary feasibility testing of a newly developed, and locally-relevant, childhood obesity treatment program for a medically-underserved community experiencing health disparities. The paper details the systems-based and CBPR processes used to engage local organizations to form a community advisory board (POPS-CAB) that identified and reviewed 3 candidate childhood obesity treatment programs, collectively rated the programs, and selected one for local adaption. This is followed by a description of a newly adapted program, *i*Choose, and the results of pilot testing the *i*Choose program in cohorts of families. The process of incorporating feedback from families and POPS-CAB members and transitioning from researcher-led implementation to community-led intervention is also described.

## Methods

### Conceptual framework and study design

The Partnering for Obesity Planning and Sustainability-Community Advisory Board (POPS-CAB) was developed in the Dan River Region (DRR) of south central Virginia and north central North Carolina [38]. Specifically, from a CBPR perspective, members of the POPS-CAB were involved in all aspects of the research from proposal development through the interpretation of study findings [39]. From a horizontal systems-based perspective, community organizations that had a primary mission to provide services to youth or families were invited to participate in the POPS-CAB with the Pittsylvania/Danville Health District, Children’s Healthcare Center, Danville Parks & Recreation, and Boys & Girls Club of the Danville Area engaging in the partnership. Further, each organization was asked to include a member(s) of the organization that engaged with youth and their families as well as an administrative member(s) that could allocate resources to develop a sustainable childhood obesity treatment option in the region (*n* = 8 community members). Research partners in the POPS-CAB had expertise in childhood obesity treatment, nutrition and exercise, health economics, implementation science, and community capacity development (*n* = 7 research members). An external consultant facilitated all POPS-CAB meetings to ensure that there was equal input by community and academic partners across the planning process, including the intervention selection process. The RE-AIM framework was used across planning and evaluation activities [40]. All study procedures were approved by the Virginia Tech Institutional Review Board. POPS-CAB members provided written informed consent for program selection and adaptation activities and parents provided written informed consent and children provided written assent prior to enrolling in the pilot trial.

This intervention development and pilot testing study is part of a larger 3-year, iterative project with two primary goals developed by the POPS-CAB. The first goal was to develop and assess community capacity related to the development, implementation, and sustainability of a local childhood obesity treatment program [39]. The second goal, addressed specifically in this paper, was to identify, adapt, implement and evaluate a locally-relevant childhood obesity program in the medically-underserved, DRR. To address its second goal, the POPS-CAB used the RE-AIM framework [40] to plan intervention strategies and operationalize program outcomes. Data were collected as part of the intervention selection process and during a 3-cohort, quasi-experimental trial of an adapted, evidence-based childhood obesity program, with a focus on acceptability and feasibility of the new program designed for local implementation.

### Intervention selection and adaptation

The POPS CAB met for a daylong meeting once a month for 3 months to develop a shared vision for the project as well as identify and select an evidence-based program for implementation in the region using the National Cancer Institute’s “Using What Works – Guide to Choosing an Evidence Based Program” process [36, 41]. This protocol for identifying, and potentially adapting, evidence-based programs includes five modules which are reported in this manuscript. The modules include [1] defining evidence-based programs, [2] assessing resources and need in the community, [3] choosing an evidence-based program, [4] adapting and summarizing the program, and [5] evaluating the program.

The research team used a multistep approach to identify potential paediatric obesity treatment programs, starting with a review of the available literature up to February 2013. Potential programs needed to have been (a) tested in multiple published studies across a range of samples, (b) provided data on efficacy and maintenance results for a period-of-time after the intervention had been completed, and (c) varied in the resources needed for implementation. Four pediatric obesity treatment interventions met these criteria--Traffic Light Diet [42–44], Bright Bodies [9, 10, 12], Golan and colleagues Home Environmental Change Model [6, 7, 45, 46], and Obeldicks [4, 5]. The Obeldicks program was excluded because there were no English (only German) program materials available at the time of the study.

For each of the three remaining programs, the following information was presented to the POPS-CAB during their daylong working meetings: [1] who delivered the program, [2] program characteristics (content, duration, number of sessions), [3] the characteristics of the participants (i.e., demographics of those who participated), and [4] available program materials and components. Because all three interventions were resource intensive and included a large number of in-person meetings over a 6–12 month period, Family Connections, an adaptation of Golan’s Home Environmental Change Model delivered with 2 small group parent sessions followed by 10 automated telephone counselling calls was included as a lower resource option with evidence of efficacy [46]. The automated telephone support calls were developed by a team with expertise in childhood obesity treatment and used branching logic based on participant responses to a set of predetermined questions and prompts [46].

In a second meeting, the POPS-CAB community partners each presented on the resources that were available to support a childhood obesity program in the region. Resources were identified that included access to the target population of high need families, personnel that could provide program sessions for families, and physical resources available to support a childhood obesity program. Specifically, both the Health District and Children’s Healthcare Center reported seeing a high proportion of low-income families while also documenting height and weight in a high proportion of children who had clinical visits. Parks and Recreation and the Boys and Girls clubs presented several physical (e.g., multiple community buildings and gymnasium space that could be used to house a childhood obesity program) and human resources (e.g., youth leaders with training in positive youth development) that currently engage youth in physical activity and other recreation pursuits.

During the third meeting, the POPS-CAB revisited the definition of evidence-based programs and reviewed findings from each of the Traffic Light, Home Environmental Change (Family Connections), and Bright Bodies Interventions using RE-AIM dimensions to characterize the potential reach, effectiveness, adoption, implementation, and maintenance information available. The objective was to evaluate these programs to ensure that the program selected could be adapted for the DRR, was appropriate for families with overweight or obese 8 to 12-year-old children, was likely to help families reduce their BMI status, could reach a large number of families that need the program, and could be adopted, implemented, and sustained in the community once grant funding was complete. The POPS-CAB also discussed modifiable aspects of evidence-based programs (e.g., changing pictures of people/places and quotes, reading level of the program materials, ways to reach your audience, incentives for participation, timeline, and cultural indicators based on the intended population) and aspects that were not considered modifiable (e.g., the health topic, deleting whole sections of the program, adding strategies that were inconsistent with the intervention model/theory, changing learning objectives).

After the review of the programs and open discussion, the POPS-CAB members received a comparison chart of the focus and components of each program (See Table 1). In addition, the program materials (e.g., workbooks, handouts, call scripts) and objectives were summarized and an example of available session materials was presented for each evidence-based program.

**Table 1:** Comparison chart of evidence-based interventions provided for POPS-CAB members

	Bright Bodies	Family Connections	Traffic Light
Nutrition			
Calorie Counting			✓
Healthy eating	✓	✓	✓
Physical Activity			
Structured Exercise	✓		
General Information		✓	✓
Topics covered			
Home environment		✓	✓
Self-monitoring	✓	✓	✓
Motivation	✓		
Self-esteem	✓	✓	
Praise and rewards		✓	✓
Role modelling		✓	✓
Goal setting	✓	✓	✓
Stimulus/cue control	✓		✓
Special occasions	✓		✓
Relapse prevention	✓	✓	
Maintenance behavior	✓	✓	✓
Lesson quizzes			✓
Delivery mode for sessions			
Individual	✓		✓
Group	✓	✓	
In-Person	✓	✓	✓
Telephone		✓	
Parent (target of session)	✓	✓	✓
Child (target of session)	✓		✓
Workbook/Resource			
Parent	✓	✓	✓
Child	✓		✓

As each of the programs had demonstrated individual level effectiveness and maintenance, the rating sheets included 16–17 items that focused on the program characteristics that the POPS-CAB had identified as important—overall program characteristics, potential for adoption, and implementation/adaptation potential. Each of the rating sheets was adapted for the specific program components (E.g., Home Environmental Change model was a parent only intervention, so ratings on child materials were not included). Ratings were based on a 5-point Likert-type scale with 1 reflecting strongly disagree and 5 reflecting strongly agree. POPS-CAB members individually rated each intervention. They were then divided into three groups with representation across community organizational and academic partners. The small groups reviewed each of the three programs, worked to develop consensus on the ratings for each program (e.g. groups completed one rating sheet for each program), and met for an additional 30 min after all programs had been reviewed to rank order the top program for each item on the rating sheet and identified the program that they considered the ‘best’ option for local testing.

Qualitative data were also collected from each of the small groups during the program rating process described above. First, to facilitate small group discussions, open-ended questions were included on the rating sheet to allow POPS-CAB members to record their perceptions about the program (i.e., “what do you like most about the program?” and “what would you most like to adapt?”). Second, each small group discussion period was audio-recorded and transcribed verbatim to provide information on the features of the childhood obesity programs that were considered when generating group ratings and rank orders. Finally, once all small groups had completed the rank order and program selection process the full POPS-CAB discussed the findings to decide on the program that would be adapted and tested locally. This discussion was also audio-taped and transcribed verbatim to provide information on areas that were discussed that contributed to the final program selection.

### Adaptation and intervention testing

Following the selection process, the POPS-CAB began the process of identifying locally available resources, potential delivery sites and personnel, and local target population characteristics (e.g., race; economic status) that should be considered for potential program adaptations. Concurrently, based on the copyright related to the selected evidence-based program materials and a desire not to inappropriately use content without permission, a curriculum subcommittee made up of research and community members of the POPS-CAB used all available research articles on the selected program [9, 10, 12, 47] to develop a set of skill-based learning objectives and principles that were covered in the original program. These learning objectives and a plan for a 3-month program duration were used to develop an adapted program called *i*Choose and materials including parent and child workbooks, biweekly family sessions, biweekly telephone support calls to parents, biweekly newsletters for children, and 3 supervised exercise sessions per week.

The POPS-CAB also identified the need to ensure that the materials were appropriate for the DRR population in regards to cultural and educational factors. As such, all materials were developed using strategies to increase the likelihood that the program would be effective for parents that may have lower health literacy levels [48]. Specifically, support calls including teach-to-goal and teach-back strategies to improve comprehension [49, 50], parent workbook materials were evaluated using clear communication strategies [51–53], and information was delivered verbally and in print. Finally, the children’s newsletter included visual and written activities that reinforced the program objectives and included pictures of participants during the exercise sessions. Table 2 provides a comparison between the original and adapted interventions. The POPS-CAB curriculum subcommittee developed lesson plans for all family and exercise sessions, power point materials to guide family sessions, and scripted guides for the telephone support calls.

**Table 2:** Comparison of inclusion criteria, structure and components of community-adapted *i*Choose program and Bright Bodies

	Bright Bodies	*i*Choose
Inclusion Criteria	• Child BMI ≥ 95th percentile • 8 to 16 years old • English-speaking • Participants have a caregiver willing to participate in the educational component	• Child BMI ≥ 85th percentile • 8 to 12 years old • English-speaking • Participants have a caregiver willing to participate in the educational component
Exclusion Criteria	• Diabetes, psychiatric disorder, or other serious medical condition • Taking medications that potentially cause significant weight gain • Taking medications for weight loss • Involved in a coexisting weight management program.	• Disabilities or conditions the exclude participation in physical activity • Non-English speaking
Duration of Program	• 12-Month Program	• 3-Month Program
Program structure	Program components included: • 80 min family sessions that would occur weekly over the first 6 months program and then biweekly for the final 6 months • Each 80-min session was divided into a nutrition lesson and a behavioral skills training • 2–50-min exercise sessions per week. All classes were child and parent together except the behavior modification topics Smart Moves Workbook supported the family session topics.	Program components included: • 90 min family sessions that would occur every other Saturday for 3 months • Each 90-min session included a nutrition lesson, behavioral skills training and exercise time • 20–25 min telephone support calls to set goals, resolve barriers, and reinforce content using 5 A’s, teach-back and teach-to-goal strategies on weeks between family sessions • 2–60-min exercise sessions per week. All classes were child and parent together except the behavior modification topics *i*Choose Workbook reinforced and supplemented optics from the family class.
Nutrition Component	• Delivered by registered dietitians. • Nondiet approach emphasizing low-fat, nutrient-dense foods and moderate portion sizes.	• Delivered by registered nurses and/or physical activity specialists. • Nondiet approach emphasizing nutrient-dense foods, sometimes foods and moderate portion size.
Physical Activity Component	• 2, 50-min Exercise Sessions per week facilitated by exercise physiologists • Each class consist of a warm-up, high-intensity aerobic exercise, and a cool-down • 100 min per week (two 50-min sessions) for the first 6 months 100 min twice per month for the last 6 months	• During the family class weeks: 1, 30-min moderate to vigorous workouts during the family class and 1 60-min moderate to vigorous workout during the week. Children and parents were encouraged to participate. • In between family classes: 2, 60-min moderate to vigorous exercise sessions per week facilitated by exercise specialists in community setting • Each class consists of a warm-up, high-intensity aerobic exercise, and a cool-down
Behavioral Modification Component	• 40-min each week for the first 6 months, every other week for 1 year facilitated by RDs or social workers • Separate classes for children and parent(s)	• 30-min every other week during the 3-month program period facilitated by RNs or physical activity specialist • Separate classes for children and parents(s)
Motivational tools	• Point system targeting regular attendance (e.g. game in which accumulated points for each exercise class or activity attended)	• Incentives/give-a-ways at each session to promote attendance • Examples included water bottles, measuring cups or t-shirts
Maintenance	• Monthly maintenance support groups for 1 year	• (none)

### iChoose program implementation process

*i*Choose was pilot tested across 3 cohorts with plans to initiate the first cohort with research staff delivery (PhD behavioural scientist; graduate research assistants), the second with co-delivery by research staff and community partner staff (Parks & Recreation Program Leaders; Public Health Nurses), and the third with community personnel (with the exception that telephone support calls were split between research and community personnel). To make the transition from research to community delivery we implemented a training structure that followed the consultee-centered protocol and included instruction on session materials, role-playing, self-evaluation, and feedback on program delivery across class, telephone, and exercise sessions [54]. Public health nurses and Parks & Recreation staff that delivered program components attended 6, 4-h training sessions led by a behavioural scientist with expertise in childhood obesity treatment programs and graduate research assistants in human nutrition, foods, and exercise. The training sessions were delivered bi-weekly and coincided with the beginning of each two-week block of the 12-week *i*Choose program. Training for the final cohort included 4, 3-h sessions that followed the same process, but spaced training out over the course of the program due to the experience of the public health nurses and Parks & Recreation staff in delivery from the second cohort.

In addition to the training protocol, across the 3 cohorts of pilot testing fidelity checklists were used to increase the likelihood of consistent implementation and to provide instructor feedback. An independent observer completed the fidelity checklists for the family sessions. The observer was unobtrusive, but, in the case that the instructor missed content, provided prompts to increase the likelihood that all content was covered. For the telephone and exercise sessions, fidelity checklists were completed by the delivery staff in real time and embedded within the call scripts or lesson plans to ensure all delivered material was captured. Fidelity checklist data were used formatively to help improve the program for each subsequent cohort and across cohorts as an indication implementation quality.

Following the first cohort, the POPS-CAB identified several changes based upon a summative evaluation completed with parents and children and notes included on the fidelity checklists for each session. Specific changes included: [1] extending the family sessions to 2 h in duration to ensure content could be covered, [2] introducing weigh-ins and family behavioural contracts at each family session, [3] introducing a primary program leader that would be present at all family and exercise sessions as well as complete the majority of the support calls, and [4] revised workbooks based on the participant feedback and clear communication index assessment by community and research partners.

### iChoose program pilot testing

The POPS-CAB used the RE-AIM framework to provide direction for initial program evaluation and identified the following outcomes for assessment: program reach and representativeness, effectiveness in reducing BMI z-scores, implementation quality for the intervention components, and 3-month, post-program maintenance of changes in BMI z-scores. Program adoption and organizational maintenance were considered in planning, but beyond the scope of assessment for this preliminary intervention development study.

Eligible children were between the ages of 8–12 years of age, BMI percentile ranking > 85%, and at least 1 caregiver willing to participate in the program. Potential participants were recruited by the health department or Children’s Healthcare Center using medical chart review to identify potentially eligible families. Nurses involved in the POPS-CAB created initial lists of potentially eligible children. Personalized letters from the child’s paediatrician were mailed to all potential families and a nurse or nurse practitioner followed the letters up with a telephone screening and recruitment call. If, after 3 attempted recruitment calls, the nurses were unable to talk to the family, research assistants made three additional attempts. Eligible and interested families were scheduled for a baseline health screening.

### Measures

#### Reach

Reach was defined as the number, proportion, and representativeness of children who assented to participate when compared to all eligible children. All parents of invited children that were contacted by telephone were asked to complete a brief screener that included gender, age, ethnicity and race, marital status, and socio-economic status related questions (e.g., highest level of parent education, annual household income, employment status, and type of health insurance).

### Implementation and adherence

Implementation fidelity of each session or telephone call was assessed based on the proportion of the key objectives that were covered during the intervention session. For physical activity sessions, the key objective was to ensure that approximately 80% of the session time was spent in moderate to vigorous physical activity. Family adherence to the intervention strategies were tracked objectively and reported as the proportion of family and exercise sessions that were attended as well as completion rates for the telephone support calls.

### Effectiveness and 3-month post-program maintenance

Effectiveness of the *i*Choose program was operationalized as change in child BMI *z*-score. Behavioural outcomes related to *i*Choose program content are reported for both child and parent participants at baseline and post-program and 3-month follow-up. Trained research staff collected anthropometric data and interview-administered surveys as each data point.

### BMI and BMI z-score

Research staff collected height and weight, without shoes and in light clothing using a calibrated digital Tanita scale (Model: 310GS) and research-grade SECA 213 portable stadiometer at baseline, post-program and 3-month follow-up on all children and parents participating in program. For children, BMI *z*-scores were computed using CDC standard age for gender algorithms and parent BMI was computed using the established kg/m^2^ formula and categorical variables created to classify parents as normal weight (BMI = 18–24.9), overweight (BMI =25–29.9) or obese (BMI > 30).

### Behavioural outcomes of physical activity, fruit and vegetable intake and sugar sweetened beverage (SSB) intake

Behavioural outcomes were measured using valid and reliable surveys for parents and children. Trained research staff interviewed administered the surveys. Minutes of moderate to vigorous physical (MVPA) activity was measured using the Godin Leisure Time Exercise Questionnaire [55]. Mean minutes of MVPA are reported. The BRFSS six-item brief screener was used to assess fruit and vegetables (FV) servings [56] and outcomes are reported as mean number of servings per day FV. Sugar sweetened beverage (SSB) consumption was measured using the Beverage Questionnaire [57, 58]. SSB is computed as mean kcals per day. Health related quality of life was measured for children using the PEDS-QL™ (children) [59]. Per PEDS-QL™ protocol, the Likert scales are converted to a percent (0–100) and higher scores indicated better health-related quality of life. Parents answered the CDC-quality days scale and this is scored and reported according to published protocols [60].

### Analysis

For the selection process, the POPS-CAB individual and group ratings were summarized and within subjects t-tests were used to determine differences in individual ratings across program characteristics, adoption likelihood, and implementation/adaptability. Though the sample size of stakeholders was small, we also compared community and academic partner ratings to determine if differences emerged in ratings. Group ratings were reported as means and final dispositions of the group rank ordering programs across rating items are presented in tabular format. The transcripts from each small group and large group discussion were reduced to meaning units (a word, phrase, or paragraph with a single meaning) and inductively categorized across the areas used to rate each intervention—we provide representative quotes within the results section indicated by quotation marks and italics.

For the intervention testing process, all data were entered by trained research assistants with a quality check completed by another research staff member. Data was entered into SPSS 22 and typical measures of central tendency are reported (mean, standard deviations, percent). Chi-square tests of association, Fisher’s exact tests and ANOVA were used to compare reach, implementation and adherence for categorical and continuous variables. Implementation and adherence data were reported by wave and across waves to provide evidence of consistency across cohorts. Due to the small cohort sizes (n = ~ 30 each) all reach, effectiveness, and maintenance data were combined for analysis across waves. Paired t-test were conducted to determine changes in weight status from baseline-post-program and 3-month-follow up- baseline to assess RE-AIM dimensions of effectiveness and maintenance. A bootstrap function was used to control for cohort and M (SE) reported for each change score.

## Results

### Intervention selection process

Following the review of program materials POPS-CAB member ratings across the 3 programs varied across perceptions of program components, adoption, and implementation features. Mean ratings were nearly identical, on average, between community and academic partners and were not statistically or practically significant—as such those data are not presented. When considering program characteristics, ratings for Bright Bodies (4.1 ± 0.4) were significantly higher than Family Connections (3.7 ± 0.4; t = 2.29, *p* < 0.05) and Traffic Light (3.7 ± 0.6; *t* = 3.48, *p* < 0.01). In contrast, Family Connections (3.8 ± 0.5) was rated significantly higher on implementation features when compared to Bright Bodies (3.6 ± 0.5; t = 1.9, *p* < 0.10) and Traffic Light (2.9 ± 0.5; t = 3.37, *p* < 0.01). Further, Bright Bodies was rated significantly higher than Traffic Light on implementation features (*t* = 2.79, *p* < 0.05). When rating adoption features the only significant difference in rating was between Bright Bodies (3.8 ± 0.7; t = 2.35, p < 0.05) and Traffic Light (3.2 ± 0.4).

When comparing the three small group ratings on program characteristics, ratings from highest to lowest included Bright Bodies, Traffic Light, and Family Connections, except for one group that rated Bright Bodies and Traffic Light the highest. In contrast, for 2 of the 3 small groups Family Connections was rated the highest on implementation and adaptation potential. In terms of adoptability, Bright Bodies was again rated the highest across all 3 small groups. Finally, the overall ratings indicated that the groups rated the programs from highest to lowest to be Bright Bodies, Family Connections, and Traffic Light. These data were considered by the small groups to rank order the top program across program characteristic, implementation/adaptation, and adoption indicators.

Data gathered from the transcripts of the small and larger group discussions were used to support the program ranking process and resulted in five consistent themes. First, POPS-CAB members expressed the importance of a program with a good balance of nutrition and physical activity content and opportunities. All groups discussed the benefits of Bright Bodies structured exercise program along with strong nutrition content. Second, there was a strong dislike across community members of the POPS-CAB related to calorie counting. This had the largest impact on Traffic Light, which was the only program that included explicit caloric restriction as part of the program. Third, there was a desire for the program to target both the parent and the child to work towards a family lifestyle change. Fourth, the POPS-CAB members reflected on the practicality and usability of the program in the target setting. Specifically, members noted that there were available resources for nutrition and behavioral counseling as well as physical activity sessions due to the combination of healthcare, public health, and youth serving organizations like Parks and Recreation and Boys and Girls club (“*As far as physical setting, none of them would be a problem, we couldn’t do it all at the health department, but with Boys and Girls club [we could].”).* Fifth, while the large group discussion led to the selection of Bright Bodies as the intervention to be delivered in the region, during that time and in the small groups, POPS-CAB members indicated that none of the three programs could be implemented without some sort of adaptation. For Bright Bodies, members identified a need to change the number of sessions (“*that’s something that we can potentially look at and say we don’t want the three times per week, let’s make it two and we can add the PA on to another the end of another session*”) and address the health literacy level of the written program materials (“*I would only be happy with major adaptations to language”).*

This ranking process and qualitative feedback by the POPS-CAB led to the decision to create the *i*Choose program, an adaptation of Bright Bodies tailored to the unique opportunities within the POPS-CAB and focused on creating a culturally relevant program for local implementation to increase likelihood of program success. Table 2 provides key distinctions between the original Bright Bodies program components and the *i*Choose program.

### iChoose recruitment, implementation and testing

#### Reach

**Fig. 1:**
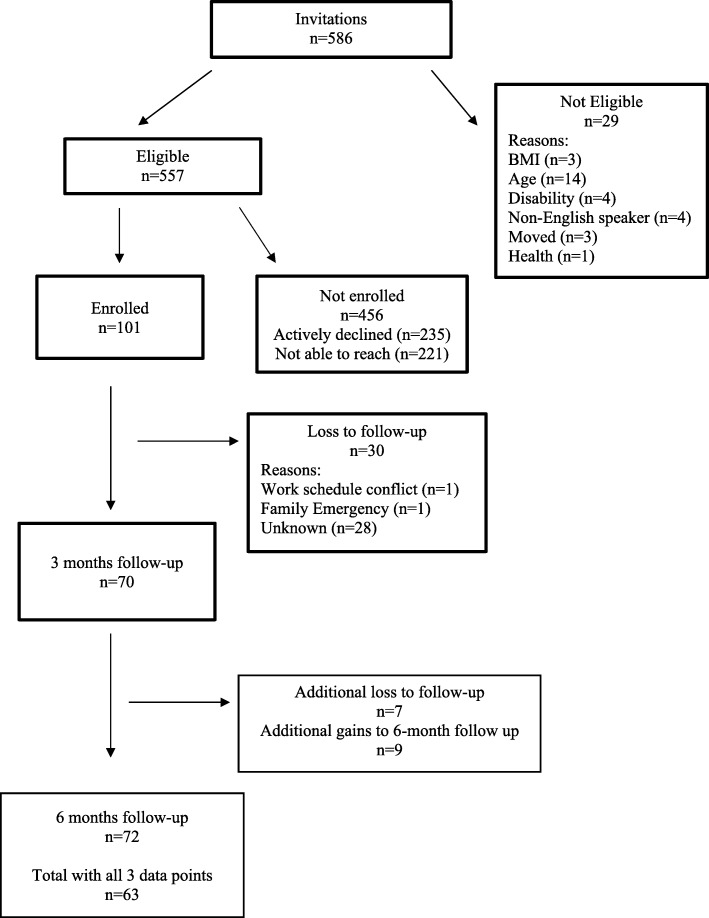
CONSORT diagram for pilot testing of *i*Choose program including all three cohorts of families

A total of 586 children were identified as potentially eligible using the medical chart reviews from the previous 6 months of visit data (Fig. 1). Physician invites were sent out to parents or guardians of all identified children and, of those contacted by telephone, *n* = 29 children were not eligible. Of the children that were identified as eligible, 18% of parents agreed to participate, 42% declined participation, and 40% were unable to be contacted after 6 call attempts over 2 weeks. When considered without families that were inaccessible by telephone, the enrollment rate was 30%, reflecting *n* = 94 families and *n* = 101 children across enrollment cohorts (Fig. 1). Table 3 provides demographic information for the representativeness of the sample enrolled compared to unenrolled children and there were no significant differences in age (10.3 vs 10.4 years), gender (52 vs 50% boys), race (61 vs 54% Black), or insurance type (71 vs 69% Medicaid).

**Table 3:** Reach and representativeness of those eligible (*n* = 557) for the *i*Choose program

	Enrolled *n* = 101	Not Enrolled *n* = 456	*p-*value
Age			
Years (Mean, SD)	10.29 (1.30)	10.44 (1.37)	.32
Gender			
Female, % (n)	48% (*n* = 49)	50% (*n* = 228)	.77
Ethnicity			
Hispanic, % (n)	3% (n = 3)	2% (n = 9)	.45
Race			
Black, % (n)	61% (*n* = 63)	54% (*n* = 246)	.38
White, % (n)	36% (*n* = 36)	43% (*n* = 196)	
Other, % (n)	1.0% (n = 1)	3% (*n* = 14)	
Health Insurance (Type)			
Medicaid, % (n)	71% (*n* = 72)	69% (*n* = 315)	.67
Private, % (n)	27% (*n* = 27)	30% (*n* = 137)	
None, % (n)	2% (n = 2)	1% (n = 4)	

#### Implementation and adherence

Table 4 provides detailed adherence and implementation quality information across intervention components and cohorts. Adherence indicators, operationalized as attendance at sessions, were highest for telephone support call completion (62% completed across cohorts), lower for family sessions (43% across cohorts), and lowest for physical activity session (33% across cohorts). Adherence was consistent across waves for support call completion (range 58–65% completed), family sessions (range 42–44% attended), and physical activity sessions (range 32–34% attended). Implementation quality, while high across all intervention components, was slightly higher for support call completion (98% fidelity) and family sessions (96% fidelity) when compared to physical activity sessions (85% fidelity). Effectiveness and 3-month post program maintenance

**Table 4:** Adherence and implementation fidelity of intervention components

	Wave 1 (n = 27)		Wave 2 (*n* = 35)		Wave 3 (*n* = 39)		Combined (n = 101)	
Family Class	Completed^*^	Fidelity	Completed^*^	Fidelity	Completed^*^	Fidelity	Completed^*^	Fidelity
Class 1	65%	94%	55%	95%	54%	100%	58%	97%
Class 2	42%	97%	42%	98%	37%	100%	41%	98%
Class 3	31%	88%	46%	98%	43%	95%	40%	92%
Class 4	42%	91%	46%	94%	37%	96%	42%	95%
Class 5	42%	93%	33%	100%	29%	98%	35%	98%
Class 6	31%	98%	46%	100%	40%	97%	39%	98%
AVERAGE	42%	93%	44%	98%	40%	98%	43%	96%
	Wave 1		Wave 2		Wave 3		Combined	
Telephone Support Calls	Completed^*^	Fidelity	Completed^*^	Fidelity	Completed^*^	Fidelity	Completed^*^	Fidelity
Class 1	65%	100%	79%	100%	77%	100%	74%	100%
Class 2	73%	96%	70%	98%	71%	100%	71%	98%
Class 3	65%	97%	70%	95%	60%	93%	65%	95%
Class 4	54%	100%	61%	99%	63%	96%	59%	98%
Class 5	46%	94%	52%	100%	51%	94%	50%	96%
Class 6	46%	94%	58%	100%	51%	100%	52%	98%
AVERAGE	58%	97%	65%	99%	63%	97%	62%	98%
	Wave 1		Wave 2		Wave 3		Combined	
PA Class	Completed^*^	Fidelity	Completed^*^	Fidelity	Completed^*^	Fidelity	Completed^*^	Fidelity
Week 1	46%	95%	42%	90%	40%	93%	43%	93%
Week 2	46%	60%	48%	98%	31%	78%	42%	79%
Week 3	40%	93%	39%	75%	36%	90%	39%	86%
Week 4	27%	70%	36%	100%	37%	75%	34%	82%
Week 5	35%	83%	33%	90%	27%	93%	32%	89%
Week 6	39%	75%	42%	90%	40%	80%	40%	82%
Week 7	15%	90%	35%	95%	30%	75%	27%	87%
Week 8	39%	80%	24%	80%	23%	80%	29%	80%
Week 9	21%	85%	24%	75%	23%	88%	23%	83%
Week 10	35%	100%	27%	90%	34%	77%	32%	89%
Week 11	19%	–	23%	83%	29%	85%	24%	84%
Week 12	23%	–	–	93%	–	93%	23%	93%
AVERAGE	32%	83%	34%	88%	32%	84%	33%	85%

^*^Percent completed is number of calls completed or number of class sessions attended based on the number of participants for each wave included in row 1. Completion and/or attendance are used to indicate overall adherence to the program

- Data not captured. For Wave 1, PA class week 11 and 12, a fidelity check list was not completed. For Wave 2 and 3, PA class in Week 12 no attendance data available

Changes in child BMI *z*-scores at post-program compared to baseline (M_Δ_ = − 0.047) were modest but significant (t = − 2.11, *p* = 0.046). This decrease in BMI *z*-scores returned to values close to baseline (M_Δ_ = 0.009) at the 3-month follow-up (Table 5). Children reported increased minutes of MVPA at post-program (M_Δ_ = 52.88 min) but this was not statistically significant. Children significantly decreased calories from SSB (M_Δ_ = − 115.44) and reported significant improvement in quality of life at the completion of the *i*Choose program (M (SE) = 3.01(1.24)). Unfortunately, these improvements were not maintained after program completion. Minutes of MVPA decreased from post-program but stayed above baseline values (non-significant). Reported kcals from SSB increased at follow-up, nearly matching baseline values. However, children continued to report increases in quality of life at the post intervention follow-up. A similar pattern of outcomes was documented for parents in the study (shown in Table 5).

**Table 5:** *i*Choose program effectiveness and maintenance information

CHILDREN								
	*Baseline* *M (SD)* *(n = 101)*	*Post-Program* *M (SD)* *(n = 71)*	*Follow-up* *M (SD)* *(n = 74)*	*Δ*_*1*_ *M (SE) (n = 71)*	*p-value*	*Δ*_*2*_ *M (SE)* *(n = 74)*	*p-value*	
BMI *z*-score	N = 101 M = 1.89 SD = 0.48	N = 71 M = 1.87 SD = 0.49	N = 74 M = 1.92 SD = 0.51	−0.047 (0.022)	**N = 71** **t = −2.11** ***p*** **= 0.046**	0.009 (0.023)	N = 74 t = 0.395 *p* = 0.672	
Physical Activity, minutes of moderate-to-vigorous physical activity	N = 91 M = 151.52 SD = 155.6	N = 64 M = 203.75 SD = 170.8	N = 71 M = 192.94 SD = 185.0	52.88 (28.6)	n = 59 t = 1.81 p = 0.063	31.35 (29.0)	*n* = 65 t = 1.09 *p* = 0.295	
Fruit and Vegetables, servings per day	N = 100 M = 3.07 SD = 2.5	N = 69 M = 3.02 SD = 2.5	N = 73 M = 3.22 SD = 2.8	−.16 (0.34)	N = 69 t = −0.44 p = 0.681	0.20 (0.34)	N = 73 t = 0.56 *p* = 0.558	
Sugar Sweetened Beverage, kcals/day	N = 100 M = 254.50 SD = 255.03	N = 69 M = 166.80 SD = 188.63	N = 73 M = 253.18 SD = 313.80	−115.44 (30.24)	**N = 69** **t = −3.67** ***p*** **= 0.001**	−4.49 (7.3)	*N* = 69 t = −0.59 *p* = 0.640	
Quality of Life, Physical^a^	N = 100 M = 74.66 SD = 15.0	N = 69 M = 75.63 SD = 12.5	*N* = 73 M = 76.05 SD = 13.8	2.49 (1.72)	N = 69 t = 1.40 *p* = 0.59	2.54 (1.74)	N = 73 t = 1.44 *p* = 0.157	
Quality of Life, Psychosocial^a^	N = 100 M = 69.50 SD = 15.5	N = 69 M = 72.68 SD = 13.8	N = 73 M = 74.09 SD = 16.9	3.29 (1.4)	N = 69 t = 2.35 *p* = .017	5.34 (1.61)	N = 73 t = 3.34 *p* = .003	
Quality of Life, Total^a^	N = 100 M = 71.29 SD = 14.0	N = 69 M = 73.71 SD = 12.3	N = 73 M = 74.77 SD = 14.9	3.01 (1.24)	**N = 69** **t = 2.32** ***p*** **= .018**	4.37 (1.43)	**N = 73** **t = 3.05** ***p*** **= .004**	
PARENTS								
	Baseline M (SD) (*n* = 94)	Post-Program M (SD) (*n* = 66)	Follow-up M (SD) (*n* = 69)	Δ_1_ M (SE) (*n* = 66)	*p*-value		Δ_2_ M (SE) (n = 69)	*p*-value
Weight, kg	N = 93 M = 98.78 SD = 25.5	N = 66 M = 98.44 SD = 26.1	N = 69 M = 98.47 SD = 25.3	−0.75 (0.29)	**N = 65** **t = −2.54** ***p*** **= .013**		.12 (0.42	*N* = 68 t = 0.27 *p* = .786
BMI, kg/m^2^	N = 93 M = 36.36 SD = 8.7	N = 66 M = 36.17 SD = 8.8	N = 69 M = 36.27 SD = 8.6	−0.28 (0.12)	**N = 65** **t = −2.23** ***p =*** **0*.*029**		−.12 (0.28)	N = 68 t = −0.44 *p* = 0.663
Physical Activity, minutes of moderate-to-vigorous physical activity	N = 92 M = 95.00 SD = 158.2	N = 61 M = 197.54 SD = 193.0	N = 67 M = 114.22 SD = 140.5	109.42 (27.06)	**N = 60** **t = 4.04** **p = 0.000**		23.91 (25.36)	N = 66 t = 0.94 *p* = 0.349
Fruit and Vegetables, servings per day	N = 94 M = 2.63 SD = 1.8	N = 65 M = 3.08 SD = 2.2	N = 69 M = 2.98 SD = 1.9	0.58 (0.19)	**N = 64** **t = 2.92** ***p*** **= 0.005**		0.32 (0.20)	N = 68 t = 1.58 *p* = 0.118
Sugar Sweetened Beverage, kcals/day	N = 94 M = 305.26 SD = 333.65	N = 65 M = 156.29 SD = 172.10	N = 69 M = 157.34 SD = 164.83	−92.91 (28.60)	**N = 65** **t = −3.26** ***p*** **= 0.002**		−119.99 (32.67)	**N = 69** **t = −3.63** ***p*** **= .001**
Health-related Quality of Life, # of unhealthy days (in last 30 days)	N = 92 M = 13.04 SD = 11.3	N = 65 M = 10.27 SD = 10.1	N = 67 M = 13.54 SD = 11.3	−1.69 (1.08)	N = 63 t = −1.56 *p* = 0.124		1.55 (1.27)	N = 64 t = 1.22 *p* = 0.225

Δ_1_ Change score computed T2-Baseline

Δ_2_Change score computed T3-Baseline

^a^Unit is percent (0–100), higher scores indicated better health-related quality of life

## Discussion

The intent of this paper was to provide a description of a process that combines a systems-based structure within a community-based participatory program of research with a goal to develop a sustainable childhood obesity treatment program in a medically underserved region. We demonstrate that this process successfully led to the selection of an evidence-based childhood obesity program, functional adaptation to that program based on evidence-based principles and learning objectives to align with and fit local resources, and preliminary evidence of effectiveness. In addition, we documented that implementation fidelity was very high across intervention sessions in-person and by telephone and was not reduced when community leaders led the family sessions or completed intervention telephone support calls. Conversely, our pilot study also indicated that the magnitude of reduction of child BMI *z*-scores was smaller than that achieved in efficacy trials [2–7, 9, 10, 12] and that the reduction was not sustained post program—suggesting additional adaptations are necessary to improve effectiveness and maintenance.

Despite the availability of efficacious interventions for treating childhood obesity, these programs continue to be underused and remain primarily located in urban areas and associated with large medical centers. There is very little evidence that these programs have been systematically translated into practice, particularly in rural areas where the clinical and professional resources needed to deliver paediatric obesity treatment programs may not exist. A leading reason for underutilization of evidence-based programs is that community organizations and healthcare systems often lack the capacity to select, adapt, and implement these programs [36, 37]. In particular, the complexity and uncertainty of the evidence-base are hypothesized to influence decision-making [36]. To address this concern, we included key stakeholders in the intervention selection process where, historically, they have not been engaged [61]. We also used the National Cancer Institute’s “Using What Works” process which we adapted to include RE-AIM information on a menu of evidence-based programs to provide guidance for CBPR initiatives planning to address areas where a strong evidence-base exists. We found that this process allowed for a consideration of not only the characteristics of an evidence-base program, but also the available resources for implementation and sustainability of that intervention by local partners [62]. We also expanded on the “Using What Works” process and included the provision of program materials for community advisory board members to review and a process to elicit partner ratings across program characteristics, adoption factors, and implementation features. This individual rating, small group rating and discussion, and community advisory board discussion of ratings was acceptable to the advisory board members and successful in assisting the board to match local resources to available evidence-based interventions.

It is important to note, the three interventions reviewed by the community advisory board varied on the type of intervention materials that were available and this may have had a large influence on the ratings and intervention selection [36]. For example, Epstein’s materials were available in a published book—the Stop-Light Diet for Children—that community advisory board members identified as potentially challenging for the local population in terms of concepts and literacy. In contrast, Bright Bodies included a workbook for families that included pictures, examples, and activities that the advisory board found to be engaging—though literacy level issues were still raised. This suggests that more research is necessary around the packaging of evidence-based materials for easy review and use by the organizations and audiences that are intended to use and benefit from the materials.

Our findings align with the proposition that using a systems-based approach that involves partners at multiple levels (e.g., delivery agent & administrator) from across community organizations will result in the selection of an evidence-based intervention that has a strong fit with local values and resources [19, 63]. This suggests that while program intensity and complexity is important in the adoption decision-making process, the degree to which it will influence the adoption decision is dependent on the systems and services involved in the collaborative [19, 36]. Specifically, the inclusion of Danville Parks and Recreation on the community advisory board resulted in a strong sense that an intervention that included multiple physical activity sessions each week could be successfully adopted, implemented and sustained. Similarly, a recent community-clinic partnership in North Carolina found that local parks and recreation staff and facilities were well equipped and prepared to deliver childhood obesity treatment programming to enrolled families [64]. Parks and recreation departments, which exist in small and large communities throughout the United States, could be a key-partners for paediatric obesity treatment efforts.

We found the adapted *i*Choose program could be delivered with high-fidelity by local partners who had received support and training from the research staff. With support, local health departments and parks and recreation professionals may be an important and underutilized partner for childhood obesity treatment programs in geographic areas where the full range of clinical expertise is not available. Key to this partnership was ongoing meetings and investment by both clinical and community partners using locally available resources and aligning intervention strategies with local community organization missions. In addition to local Parks and recreation partners, the public health department and public health nurses proved key partners in program delivery.

The *i*Choose program resulted in modest, but significant reduction in BMI *z*-score for children. However, these changes were not maintained at three months. There are several possible explanations for why our results differ from the original Bright Bodies studies [9]. First, there could be differences in the sample recruited, in the exclusion criteria between the trials and the shorter duration of the program (3 months versus 6 to 12 months). Second, the expertise of the personnel delivering the intervention, implementation fidelity, or adaptations that were inconsistent with the principles that underlie Bright Bodies. However, our focus on capturing process data reduce the likelihood that the lower magnitude of effect is due to implementation as our data show that intervention delivery staff implemented the intervention with a high degree of fidelity, regardless of personnel expertise.

As pragmatic trial, our recruitment strategies were developed to recruit a representative sample of participants in a low income and medically-underserved area. As such, we did not include a run-in period to exclude less motivated families, we rescheduled families who did not show up for initial assessments, and used financial incentives for study assessments that may have encouraged families to enroll in the study simply to receive the incentives which were not tied to participation in the intervention sessions [65]. Thus, the representativeness of families, which is documented in our data, may also include representativeness across participant motivation to engage in the intervention with some motivated and others not. This is a primary critique of expecting the same magnitude of effect from efficacy and effectiveness studies [66] and our findings seem to support this in that approximately 42% of the participants did not attend a family intervention session and approximately 26% of parent participants never completed a telephone support call. When considered within the context of a community with limited resources for childhood obesity treatment, it may be prudent to incorporate methods to improve motivation prior to enrollment (e.g., motivational interviewing) and include strategies to identify families that are most likely to engage in the intervention.

For pilot testing purposes, the *i*Choose program was 3-months in duration, which may not be long enough to generate larger effects and an active maintenance phase could extend positive program impacts [67]. For example, during the *i*Choose program, participating children and parents could attend twice weekly instructor-led physical activity sessions designed to achieve moderate-to-vigorous intensity physical activity. This increased MPVA for children and parents during the program, but was that increase was not sustained at 3-months. Offering ongoing *i*Choose specific physical activity programming may provide a much-needed opportunity for children to participate in meaningful physical activity. Importantly, children reported sustained increases in quality of life after the completion of *i*Choose programming suggesting a sustained positive impact for the children. Parents self-reported quality of life improved during the program but returned to baseline values at 3-months follow-up. Parents may benefit from ongoing support of family sessions to meet challenges of making these family-based changes.

Somewhat related, is the question of adaptations to the program. The process we used focused explicitly on the underlying principles and learning objectives of Bright Bodies and was very flexible in changing the structure in terms of number of in-person sessions, shifting parent support to telephone calls, and shortening the program without maintenance sessions after the program core was delivered. Based on family feedback and the lack of maintained effects, it appears that the shift to a 3-month program without additional booster sessions was problematic. However, POPS-CAB community stakeholders also suggested that longer programs would lead to more difficulty in recruiting and sustaining participation. Recommendations from our stakeholders were to focus on previous, successful program participants as potential role models and support networks for new cohorts of participants and to extend the program using technology approaches that would reduce the ‘class attendance burden’.

Some overarching limitations to this work should be noted. First, this study was designed as a feasibility pilot trial of the newly adapted *i*Choose program, thus we do not use a control condition for comparison. This does reduce our internal validity. However, we aim to reduce potential biases by using an objective measure (BMI *z*-score) for our primary outcome. Additionally, the overarching goal of this study was to adapt an effective obesity treatment program for local implementation; thus, we began with programs that have already been shown to be effective. The selected program, Bright Bodies has demonstrated effectiveness in other studies establishing internal validity. We also focus on pragmatic factors related to external validity and the changes in outcomes when delivering an effective program in ‘real-world’ settings. As a feasibility trial, the sample size was modest and with program attrition, our final sample was *n* = 71. This attrition rate falls within the range (5–46%) reported in a meta-analysis of paediatric obesity treatment programs [68] and at the higher ends of the ranges reported in previous trials of Bright Bodies, Traffic Light, and Golan’s Home Environmental Change model (range 3–30%) [68]. We opted to conduct analysis on the completers rather than imputations from baseline (baseline-carried-forward) or end-of-program (last-value-carry forward); both of which may have led to an overly positive interpretation of our primary outcome, reduction or no change to child BMI *z*-scores at end of program and during maintenance. Analysing the completers only allows us to more precisely understand the program influences on our primary outcome at the end of program and what turned out to be a lack of sustained effects, both important outcomes to consider for a larger trial. As noted above, using methods to enhance retention of program participants and to extend programming or support to a longer time frame are important considerations for future iterations of the *i*Choose program.

## Conclusions

While the area of implementation science has gained traction over the past decade, few studies report on the adoption process by which organizations select, adapt, and test an evidence-based program for local delivery. The process and strategies related to initial program identification and selection are distinct from the training and resource deployment that are necessary for initial uptake, adaptation, and implementation—and we provide a process for other community-engaged researchers to follow. Specifically, the process used in our project provides a demonstration of a guided evidence-based program selection process that acknowledges the existing evidence base concurrently with the assessment of available community resources that could be used for on-going implementation—and is likely generalizable to any number of systems attempting to select appropriate evidence-based interventions for implementation. Further, the adaptation and training processes used resulted in high quality implementation across intervention sessions and are likely generalizable to other communities [54]. The findings for reach, effectiveness, and maintenance were less encouraging and suggest that addressing recruitment and motivation early is especially important for pragmatic trials intent on engaging a representative sample of high-need families. In addition, our participatory approach has led to successful funding and execution for the next phases of effectiveness and implementation research using stakeholder- and patient-centered approaches. Adaptations have been made to increase the likelihood of maintained effects, including extension of the program with low-burden strategies and the use of previous, successful program participants as a social network for new program participants.

## Abbreviations

BMI: Body mass index
CBPR: Community based participatory research
DRR: Dan River Region
EBP: Evidence based programs
FV: Fruit and vegetable
HEM: Home environmental model
kcals: Kilocalories
MVPA: Moderate-to-vigorous physical activity
PA: Physical activity
POPS: Partnering for Obesity Planning and Sustainability
POPS-CAB: Community advisory board
SSB: Sugar Sweetened Beverage
